# Validation of the Spanish version of the Multidimensional State Boredom Scale (MSBS)

**DOI:** 10.1186/s12955-015-0252-2

**Published:** 2015-05-15

**Authors:** Marta Alda, Joaquin Minguez, Jesús Montero-Marin, Margalida Gili, Marta Puebla-Guedea, Paola Herrera-Mercadal, Mayte Navarro-Gil, Javier Garcia-Campayo

**Affiliations:** Instituto Aragonés de Ciencias de la Salud, Miguel Servet Hospital and University of Zaragoza, Primary Care Prevention and Health Promotion Research Network (RedIAPP), Zaragoza, Spain; University of Zaragoza, Zaragoza, Spain; Faculty of Health and Sport Sciences, Huesca, Spain; Primary Care Prevention and Health Promotion Research Network (RedIAPP), Zaragoza, Spain; Institut Universitari d’Investigació en Ciències de la Salut (IUNICS), University of Balearic Islands, Mallorca, Spain

**Keywords:** Boredom, Questionnaire, Validation, Multidimensional State Boredom Scale

## Abstract

**Background:**

Boredom, which is a common problem in the general population, has been associated with several psychiatric disorders. The Multidimensional State Boredom Scale (MSBS) was developed, based on a theoretically and empirically grounded definition of boredom, to assess this construct. The aim of the present study was to assess the psychometric properties of the Spanish validated version of the MSBS in a multi-age sample recruited from the general population.

**Methods:**

The patients (N = 303) were recruited from primary care settings. In addition to the sociodemographic variables and the MSBS, the General Health Questionnaire 28 items (GHQ-28), Positive and Negative Affect Scale (PANAS), Negative subscale and the Mindful Attention Awareness Scale (MAAS) were administered. We used confirmatory factor analysis (CFA) to analyse the dimensionality of the MSBS. Cronbach’s α coefficient was used to analyse the internal consistency of the scale. The consistency of the MSBS over time (test-retest reliability) was assessed using the intra-class correlation coefficient. The construct validity was examined by calculating Pearson’s *r* correlations between the MSBS with theoretically related and unrelated constructs. Cronbach’s α for MSBS was 0.89 (95 % CI, 0.87–0.92), ranging from 0.75 to 0.83 for the 5 subscales.

**Results:**

The characteristics of the final sample (N = 303) were that the participants were primarily female (66.77 %) with a mean age of 49.32 years (SD, 11.46) and primarily European (94.71 %). The CFA of the MSBS confirmed that the original five-factor model showed good fit indices: CFI = .96; GFI = .94; SRMR = .05; and RMSEA = .06 [.05–.08]. Cronbach’s α for MSBS was 0.89 (95 % CI, 0.87–0.92), ranging from 0.75 to 0.83 for the 5 subscales. The MSBS showed a test-retest coefficient measured with an ICC of 0.90 (95 % CI, 0.88–0.92). The ICC for the 5 subscales ranged from 0.81 to 0.89. The MSBS showed a significant negative correlation with MAAS and a significant positive correlation with the GHQ (total score and subscales) and PANAS-Negative Affect.

**Conclusions:**

The Spanish version of the MSBS has been validated as a reliable instrument for measuring boredom in the general population. This study will facilitate the assessment of boredom for clinical and research purposes in Spanish-speaking populations.

## Background

Boredom can be defined as “the experience of being disengaged from the world and stuck in a dissatisfying present” [1]. It is a common problem: in a survey of North American youth, 91 % of the respondents reported that they experience boredom [2]. It has been associated with several psychiatric disorders, such as depression and anxiety [3], somatisation [4], overeating and binge eating [5], pathological gambling [6], alcohol abuse [7] and marijuana use [8]. Additionally, boredom has been associated with a decrease in psychological well-being, such as lowered levels of life meaning [9], life satisfaction [3] and job satisfaction [10]. Finally, boredom is even associated with mortality, giving support to the popular phrase “bored to death” [11].

There are several explanatory theories on boredom [1, 12]. They support a multidimensional concept of boredom that includes (a) lack of engagement, (b) low arousal negative affect, (c) high arousal negative affect, (d) the experience of a slow passage of time, and (e) difficulty focusing attention [1].

Several scales can be used to assess boredom; however, they are either subfactors of scales measuring other constructs or are very narrow in scope (i.e., they evaluate boredom in only one particular context, such as leisure time or sexual relationships) [1]. The only scale that is more widely used to measure this construct, i.e., the Boredom Proneness Scale [3], assesses one’s tendency to become bored (trait boredom) and does not assess the actual experience of boredom in a given moment (state boredom) [1].

A new scale, the Multidimensional State Boredom Scale (MSBS), was developed based on a theoretically and empirically grounded definition of boredom and was validated [1]. This scale shows a five-factor structure, with adequate psychometric measures that positively correlate with depression, anxiety, impulsivity and neuroticism and negatively correlate with life satisfaction and purpose in life [1]. The aim of the present study was to assess the psychometric properties of the Spanish validated version of the MSBS in a multi-age sample recruited from a primary care setting.

## Methods

### Design

Validation study.

### Participants

The participants were recruited from a primary care setting within a large study on the efficacy of computer-assisted psychotherapy [13]. The following inclusion criteria were used: individuals ranging in age from 18 to 65 years and who agreed to participate in this study. The exclusion criteria were any medical or psychiatric disorders that would impede the individual from answering the questionnaire correctly and poor knowledge of the Spanish language. The sample size was calculated according to the recommended 10:1 ratio for the number of subjects to the number of test items [14]. The questionnaires and protocols used in this study were approved by the Ethical Committee of the regional health authority, and the patients signed a consent form attesting to their willingness to participate in this study. The study was conducted between September 2013 and June 2014.

### Measures

#### Sociodemographic variables

Background information from the participants included age, gender and level of education (primary school, secondary school, and university).

#### Multidimensional State Boredom Scale (MSBS)

This is a self-reported 29-item scale with a five-factor structure: disengagement, high arousal, low arousal, inattention and time perception. This is the only full scale measure of state boredom. It presents adequate psychometric properties [11], and it is the questionnaire to be validated.

#### The General Health Questionnaire (GHQ)

It is a screening device for identifying minor psychiatric disorders in the general population and within the community or non-psychiatric clinical settings, such as primary care or general medical out-patient settings. It has several versions (60, 30, 28 and 12 items) [15]. The 28-item version includes 4 subscales: somatic symptoms, anxiety and insomnia, social dysfunction and depression. The GHQ-28 has been validated in Spanish [16].

#### Positive and Negative Affect Schedule

The PANAS [17] is a brief measure of positive and negative affect. The PANAS consists of a list of 20 adjectives (10 per subscale) rated on a 5-point scale using the time instructions desired by the researcher. Present moment instructions were used in the present study. This questionnaire has been validated in Spanish [18].

#### The Mindful Attention Awareness Scale (MAAS)

The MAAS [19] is a 15-item measure of mindfulness. The item content was designed to reflect the opposite of the construct of mindfulness, or “mindlessness,” and thus endorsing the item content at a lower frequency is perceived to mean a higher level of mindfulness. Each item is rated on a scale from 1 (almost always) to 6 (almost never) in relation to the respondent’s “everyday experience,” and there is no specified time frame for these ratings. The item ratings are averaged to form the total score. The scale has been recently validated in Spanish showing appropriate psychometric parameters [20].

MAAS and GHQ were included because boredom proneness has been demonstrated to negatively correlate with mindfulness and positively with anxiety and depression [21]. PANAS was included in the original validation [1] because it positively correlates with Negative Affect and negatively correlates with Positive Affect.

### Validation process

Permission to translate and validate the MSBS was obtained from the original authors [1]. Two researchers, who were aware of the questionnaire’s objectives, performed the initial translation into Spanish. Each researcher translated the questionnaire separately. Subsequently, two bilingual linguistic experts, who had no specific knowledge regarding the instrument, carried out back-translations. Finally, the two English versions were determined to be equivalent by a native English-speaking English teacher. Any differences between the translations were resolved by mutual agreement. The usual guidelines have been followed for cross-cultural adaptations [22]. The final Spanish version is shown in Annex 1. Assessments took place at two different points over a 1- to 2-week interval. The subsample for the second assessment was randomly selected.

### Statistical analysis

The demographic data were analysed using the descriptive statistics of mean, standard deviation (SD) and range. Before conducting the statistical analyses, we examined the data for univariate and multivariate outliers. To detect the presence of univariate outliers, the frequency distributions of each item were examined (values ≥ 3 standard deviations from the mean indicate univariate outliers). The multivariate outliers were screened using the Mahalanobis distance scores for all cases (D2). A D2 probability ≤ 0.01 indicates the existence of multivariate outliers [23]. We eliminated 3 participants who were considered to be outliers according to a given scale.

We used the confirmatory factor analysis (CFA) to analyse the dimensionality of the MSBS. We proposed the previously described five-factor model [1]. EQS software for Windows version 6.1 [24] was used to conduct the CFA. The maximum likelihood with a robust correction method was used to adjust for distributional problems in the data set. Although a model with a non-significant chi-square estimate is generally considered to be a model with good fit, Hu and Bentler [24] recommended combinational rules to evaluate the model fit. Therefore, we analysed the following indices (values in parentheses denote goodness-of-fit standards): Comparative Fit Index and Goodness of Fit Index (CFI and GFI > .90) and Root Mean Square Error of Approximation (RMSEA) and Standardised Root Mean-Square Residual (SRMR < .08) [24]. We selected these statistics to measure the fit because previous studies have validated the performance and stability of these tests [25].

We examined the internal consistency, test-retest and construct validity of the MSBS. Cronbach’s α coefficient [26] was used to analyse the internal consistency of the scale. Corrected item-total correlations, in which an item is correlated with the total scale score, excluding itself, were tested for each item. The consistency of the MSBS total score over time (test-retest reliability) was assessed using the intra-class correlation coefficient (ICC). The construct validity was examined by correlating the MSBS with theoretically related and unrelated constructs. Pearson’s *r* correlations were performed to evaluate the univariate relationships between the MSBS and the following variables: psychological distress, negative affect and mindfulness. All of the statistical analyses, except CFA, were performed using SPSS software, Release 19 (SPSS Inc., Chicago, IL, USA).

## Results

### Characteristics of the sample

A total of 311 patients from primary care settings in the city of Zaragoza were recruited. Of these 311 patients, 4 (1.27 %) patients refused to participate, 1 patient (0.31 %) was ruled out because of a severe medical condition that made it difficult to answer the questionnaires (dementia) and 3 (0.96 %) were not fluent in Spanish. The characteristics of the final sample (N = 303) were that the participants were primarily female (66.77 %) with a mean age of 49.32 years (SD, 11.46; range, 19–67 years) and primarily European (94.71 %). There was no association between age and MSBS (Pearson: −0.183; p = 0.32).

### Confirmatory factor analysis

All of the items were examined in terms of mean, standard deviation, skewness and kurtosis. On the basis of these values, all of the data showed normality. The CFA of the MSBS confirmed that the original five-factor model [1] showed good fit indices: CFI = .96; GFI = .94; SRMR = .05; RMSEA = .06 [.05–.08]. The factor loadings of the 29 items of the questionnaire are summarised in Table 1.

**Table 1:** Confirmatory factor analysis. Loading of ítems of the MSBS (N = 303)

	Factors				
Item test	DIS	HA	LA	IN	TP
DIS					
22. I am wasting time that would be better spent on something else	**.59**	.10	.03	.11	.07
28. I feel like I’m sitting around waiting for something to happen	**.55**	.08	.12	.24	.08
2. I am stuck in a situation that I feel is irrelevant	**.47**	.21	.13	.03	.11
7. Everything seems repetitive and routine to me	**.44**	.17	-.03	-.06	.10
9. I seem to be forced to do things that have no value to me	**.40**	.12	.09	.14	.15
24. I want something to happen but I’m not sure what	**.37**	-.05	.14	.07	.05
10. I feel bored	**.34**	.15	.10	.08	.07
19. I wish I was doing something more exciting	**.33**	.04	.15	.11	.08
13. I am indecisive or unsure of what to do next	**.32**	.10	.03	.25	.12
17. I want to do something fun, but nothing appeals to me	**.31**	.09	.09	.12	.27
HA					
5. Everything seems to be irritating me right now	.12	**.67**	.02	.03	.12
14. I feel agitated	.11	**62**	-.02	.08	.09
12. I am more moody than usual	.02	**.55**	.23	.06	.04
27. I am annoyed with the people around me	-.14	**.51**	.11	.03	.24
21. I am impatient right now	.22	**.46**	-.02	.18	.03
LA					
4. I am lonely	.04	.02	**.70**	.09	.05
15. I feel empty	.12	-.03	**.65**	.11	.08
25. I feel cut off from the rest of the world	.08	.04	**.61**	.07	.09
29. It seems like there’s no one around for me to talk to	.06	-.11	**.55**	.09	.14
8. I feel down	.07	.22	**.48**	.07	.11
IN					
16. It is difficult to focus my attention	.02	.08	.07	**.61**	.08
3. I am easily distracted	.04	.09	-.11	**.54**	.07
23. My mind is wandering	.08	.12	.09	**.51**	.12
20. My attention span is shorter than usual	.10	.09	.12	**.45**	.19
TP					
1. Time is passing by slower than usual	.08	-.11	.09	.04	**.56**
6. I wish time would go by faster	.12	.08	.15	.08	**.51**
18 . Time is moving very slowly	.11	.07	.03	.18	**.45**
11. Time is dragging on	.05	.09	.07	.14	**.41**
26. Right now it seems like time is passing slowly	.14	.08	.11	.07	**.38**

*DIS* disengagement, *HA* high arousal, *LA* low arousal, *IN* inattention, *TP* time perception

Values in boldface represent salient items with regard to that factor

To replicate the findings of the original authors, a second-order model was assessed and showed good fit indices: CFI = .95; GFI: 0.93; SRMR = 0.5; RMSEA = 0.7 [0.6–0.8]. The loadings of the first-order factors on the second-order factors were as follows: .91 for DIS, .83 for HA, .85 for LA, .80 for IN and .64 for TP. It confirms that the MSBS measures five specific factors that combine to form a single general construct of boredom.

### Internal consistency and test-retest reliability

Cronbach’s α for MSBS was 0.89 (95 % CI = 0.87–0.92), ranging from 0.75 to 0.83 for the 5 subscales. All corrected item-total *r* correlation coefficients were above 0.30: the scoring ranged between 0.39 and 0.69. These data indicate a high degree of internal consistency for MSBS (Table 2). With regard to temporal stability, a subsample of 123 (40.59 %) individuals was randomly selected and a new interview was arranged for 1–2 weeks later. In this subsample, 62.60 % were female, the mean age was 46.87 years (SD, 9.65), and 97.56 % were European. There were no significant differences in the sociodemographic variables between this subsample and the entire sample. In this subsample, the MSBS showed a test-retest coefficient measured with an ICC of 0.90 (95 % CI, 0.88–0.92). The ICC for the five subscales ranged from 0.81 to 0.89 (Table 2).

**Table 2:** Internal consistency and test-retest reliability of the subscales and total MSBS score

MSBS	Internal consistency	Test-retest reliability
	(Cronbach’s alpha)	(ICC)
Disengagement	.83	.89
High arousal	.80	.83
Low arousal	.81	.87
Inattention	.75	.81
Time perception	.78	.85
Total MSBS score	.89	.90

### Construct validity

To test the construct validity, Pearson’s correlation coefficients were calculated between the MSBS and other questionnaires measuring related constructs. The studied constructs follow a normal distribution. As expected, the MSBS showed a significant negative correlation with MAAS and PANAS-Positive Affect and a significant positive correlation with the GHQ (total score and subscales) and PANAS-Negative Affect (Table 3).

**Table 3:** Correlation between MSBS and other psychological variables

Questionnaires	MSBS
MAAS	-.36*
GHQ global	.39*
GHQ somatic symptoms	.38*
GHQ anxiety and insomnia	.41*
GHQ social dysfunction	.42*
GHQ depression	.46*
PANAS-Positive subscale	-.43*
PANAS-Negative subscale	.49*

*p < 0.001

## Discussion

The primary purpose of the present study was to validate the Spanish version of the MSBS. To the best of our knowledge, despite the importance of the boredom construct, there is no available validation of any questionnaire for assessing MSBS in Spanish.

In our study, the MSBS factorial structure observed using CFA was largely consistent with that reported by the original authors [1]: the five-factor model showed adequate fit and all of the items loaded strongly onto the expected latent factor. The maintenance of the factor structure cross-culturally could be expected because the process of development of the scale was exhaustive from a methodological viewpoint. Additionally, MSBS showed high internal consistency and high test-retest reliability. The high test-retest reliability found in this study may be surprising for a state measure. However, the developers of the scale [1], acknowledged that MSBS to be a potentially foundational tool for the study of both state boredom and boredom proneness. Test-retest reliability was assessed at 1–2 weeks interval without any kind of psychological intervention in this period. It is likely that boredom-prone individuals need more time than 1–2 weeks for assessing changes in boredom levels in their outer world. Future studies should include retest at different intervals to answer this question.

Despite clinical experience and popular thought suggesting that state boredom is more frequent in the elderly, no association was found between state boredom and age. More specific research to assess this question could be necessary, by means of invariance factor analysis, using structural equation modelling, and comparing the structure of the questionnaire and factor loadings between different age ranges.

Finally, expected and significant correlations with other related psychological variables were observed: MSBS inversely correlates with mindfulness (measured by MAAS) and positive affectivity (measured by PANAS) and positively correlates with negative affectivity (PANAS-Negative affect) and psychological disturbance (GHQ-28-global and its four subscales: somatic symptoms, anxiety and insomnia, social dysfunction and depression).

Recent studies have demonstrated [27] that there are differences between cultures, specifically between European Canadians and Chinese, in state boredom levels, with these being higher in European Canadians. This is coherent with previous studies that affirm that when compared to Asians, Europeans tend to value more high-arousal positive affects (eg: excitement) and less low-arousal positive affects [28].

The primary limitations of the study are the same as described by the authors who developed the original scale [1]. First, the scale is long (N = 29 items) and uncomfortable for research or clinical purposes; however, the multidimensionality of the construct boredom requires this complexity. A shorter scale should be a research target in the future. Second, all of the items in the scale are positively keyed to avoid creating a factor structure based on direction or wording [29]; this fact facilitates social desirability answers. Third, as in any study using self-report measures, the results may have been influenced by the participants’ acquiescence and the need for social desirability.

However, compared with the original validation study, one of the strengths of the study is that the present study has been conducted in primary care settings in a multi-age sample. This sample is representative of the patients who consult healthcare services in a universal free public health system, such as in the Spanish one, and it is also representative of the general population. The conceptualisation of boredom and the development of the MSBS were conducted with a young, fairly educated adult sample (1). However, this Spanish validation has been studied in a multi-age sample; so, the MSBS seems to work in populations of any age.

## Conclusions

The Spanish version of the MSBS has been validated to be a reliable instrument for measuring boredom in the general population. Although this psychological construct is considered to be relevant for its relationship with many psychiatric disorders, there have not been many studies that enhance our knowledge of this construct and its relationship with mental health. This study will facilitate the assessment of boredom for clinical and research purposes in Spanish-speaking populations.

## Annex 1. Spanish validated version of the MSBS

Instrucciones: Responda a cada pregunta indicando cómo se siente ahora sobre sí mismo y su vida, incluso si es diferente a como se siente normalmente.

Use las siguientes opciones: 1 Muy en desacuerdo; 2 En desacuerdo; 3 Algo en desacuerdo; 4 Ni de acuerdo ni en desacuerdo; 5 Algo de acuerdo; 6 De acuerdo; y 7 Muy de acuerdo.

1. El tiempo pasa más lento de lo habitual.
2. Estoy atascado en una situación que siento que es irrelevante.
3. Me distraigo con facilidad.
4. Me siento solo.
5. Todo parece estar irritándome ahora mismo.
6. Desearía que el tiempo pasara más deprisa.
7. Todo me parece repetitivo y rutinario.
8. Me siento bajo de ánimo.
9. Parece que esté forzado a hacer cosas que no tienen valor para mí.
10. Me aburro.
11. El tiempo se me hace eterno.
12. Tengo más cambios de humor de lo habitual.
13. Estoy indeciso o inseguro sobre qué hacer después.
14. Me siento agitado.
15. Me siento vacío.
16. Me resulta difícil focalizar la atención.
17. Quiero hacer algo divertido, pero nada me atrae.
18. El tiempo transcurre muy lentamente.
19. Desearía estar haciendo algo más emocionante.
20. Mi periodo de atención es más corto de lo habitual.
21. Estoy impaciente ahora mismo.
22. Estoy desperdiciando tiempo que estaría mejor aprovechado en otra cosa.
23. Mi mente está dispersa.
24. Quiero que ocurra algo, pero no estoy seguro de qué.
25. Me siento apartado del resto del mundo.
26. Ahora mismo, parece que el tiempo está pasando lentamente.
27. Estoy enfadado con la gente de mí alrededor.
28. 28 Me siento como si estuviera de brazos cruzados esperando a que ocurra algo.
29. Parece como si no hubiera nadie alrededor con quién poder hablar.

## Abbreviations

MSBS: Multidimensional State Boredom Scale
MAAS: Mindful attention awareness scale
GHQ: General Health Questionnaire
PANAS: Positive and Negative Affect Schedule
SD: Standard deviation
CFA: Confirmatory factor analysis
ICC: Intraclass correlation coefficient
CFI: Comparative fit index
GFI: Goodness of fit index
SRMR: Root mean square error of approximation
RMSEA: Standardised root mean-square residual
